# Stroke walking and balance characteristics via principal component analysis

**DOI:** 10.1038/s41598-024-60943-5

**Published:** 2024-05-07

**Authors:** Jieun Cho, Sunghe Ha, Jooyoung Lee, Minseok Kim, Hogene Kim

**Affiliations:** 1 Translational Research Centre on Rehabilitation Robots, National Rehabilitation Centre, Ministry of Health & Welfare, Seoul, South Korea; 2 Department of Physical Education, College of Sciences in Education, Yonsei University, Seoul, Korea; 3 Department of Applied Statistics, Chung-Ang University, Seoul, South Korea; 4 Department of Mechanical Engineering, University of Michigan, Ann Arbor, MI USA

**Keywords:** Stroke, Balance impairment, Gait, Principal component analysis, Kinematics, Stroke, Predictive markers, Disability

## Abstract

Balance impairment is associated gait dysfunction with several quantitative spatiotemporal gait parameters in patients with stroke. However, the link between balance impairments and joint kinematics during walking remains unclear. Clinical assessments and gait measurements using motion analysis system was conducted in 44 stroke patients. This study utilised principal component analysis to identify key joint kinematics characteristics of patients with stroke during walking using average joint angles of pelvis and bilateral lower limbs in every gait-cycle percentile related to balance impairments. Reconstructed kinematics showed the differences in joint kinematics in both paretic and nonparetic lower limbs that can be distinguished by balance impairment, particularly in the sagittal planes during swing phase. The impaired balance group exhibited greater joint variability in both the paretic and nonparetic limbs in the sagittal plane during entire gait phase and during terminal swing phase respectively compared with those with high balance scores. This study provides a more comprehensive understanding of stroke hemiparesis gait patterns and suggests considering both nonparetic and paretic limb function, as well as bilateral coordination in clinical practice. Principal component analysis can be a useful assessment tool to distinguish differences in balance impairment and dynamic symmetry during gait in patients with stroke.

## Introduction

Asymmetric postural control is a distinguishing characteristic of individuals who have experienced a stroke. Approximately 55.5% of individuals with chronic stroke reportedly exhibit gait asymmetry^1^. Asymmetric postural control during walking results in insufficient balance control^2^, elevated energy expenditure^3^, higher risk of musculoskeletal degeneration and pain^4^, reduced level of daily activity levels^5^, and falls^6,7^. Owing to unilateral impairments in patients who have experienced a stroke, asymmetrical postural control when walking initiates a harmful chain reaction of faulty motor-sensory feedback. This causes hastened asymmetries, imbalanced body alignment, and heightened musculoskeletal problems that exacerbate improper postural control^4,5^.

Asymmetric gait is associated with poor balance control^2^. Previous studies have indicated a correlation between standing and gait asymmetry in individuals poststroke^1,2,8–11^. A recent study on 39 patients who had experienced a stroke found that Berg balance scale (BBS) scores were correlated with asymmetries in step length and swing time (r = − 0.36 to − 0.63)^2^. Other studies have found associations between quiet standing symmetry and spatiotemporal symmetry during walking (e.g., swing/stance time and step length)^1,8,9^. However, targeted gait training to address this spatiotemporal asymmetry in patients who experienced a stroke resulted in improvements in walking endurance and metabolic cost, not in asymmetrical gait parameters^11^. A longitudinal study on patients who had experienced a stroke indicated that rehabilitation did not improve most patients’ spatiotemporal asymmetry, despite improvements in gait speed, balance, and functional mobility^10^. Additionally, spatiotemporal gait symmetry, represented by step length and swing/stance time ratios of the paretic and nonparetic side, has been proposed as a gait quality and recovery measure^12,13^. However, several limitations persist concerning these gait parameters as they provide limited information from the data acquired during gait. Therefore, the multicollinearity of spatiotemporal biomechanical data should be considered using diverse approaches. This comprehension can provide a useful foundation for developing rehabilitation interventions for patients who have experienced a stroke, considering their associations between individual postural control strategies during gait and balance impairments.

Recent biomechanical studies have frequently used principal component analysis (PCA) to measure the joint coordination of body segments. PCA enables the deconstructing of complex full-body motion trajectories into sets of motion components, facilitating the identification of distinct motion characteristics across different groups and conditions^14–17^. A recent study, utilizing PCA, found that frail older adult women display greater ankle and knee joint variability in the sagittal plane while walking compared with non-frail older adult women^15^. Another PCA study found that individuals who had experienced falls had different joint kinematic characteristics than those who had not experienced falls, indicating that reducing the variability of joint kinematics can lower the risk of falling^14^. Several studies investigating post-stroke PCA have extracted independent gait characteristics from spatiotemporal, kinematic, and kinetic metrics^16,17^. These studies have suggested that PCA can provide insight into different gait pathologies and compare the progress of individuals with similar pathologies. However, the total variance of stroke PCAs is inconsistent, ranging from 63.8 to 81.9%, which is insufficient in reflecting movement characteristics^17,18^. Although balance impairment and asymmetric gait patterns are prominent and significant features of patients who have experienced a stroke, no studies have examined their relationship using PCA. Additionally, a comparison of bilateral limb differences would further enhance the understanding of postural control in strokes.

Therefore, this study aimed to examine the differences in bilateral kinematic characteristics among patients with balance impairment after a stroke using a novel PCA method. The present study is expected to be a significant advancement in comprehending the movement characteristics of bilateral lower limbs in patients who have experienced a stroke and propose individualized gait interventions based on their balance capacity in clinical practice. This study hypothesises that differences in gait kinematics exist in paretic and non-paretic limbs between patients who have experienced a stroke with balance impairment and those without. It was also hypothesized that walking in balance impaired patients who have experienced a stroke involves increased joint variability of the lower extremities during the gait cycle compared with that in those who had non-impaired balance.

## Results

### Baseline comparison

Of the 45 participants recruited for inpatient rehabilitation, 44 were included in this study. All participants were capable of walking independently for a distance of 10 m without the use of any assistive devices and did not require any such devices in their daily activities. Of these, 14 (31.8%) exhibited balance impairment and were assigned to the BBS low group. The baseline characteristics of the BBS high and BBS low groups are presented in Table 1. Significant differences were observed in range of motion of ankle dorsiflexion, isometric contraction force of ankle dorsiflexor and ankle invertor, BBS, timed up and go (TUG), fall efficacy scale, mini-mental state estimation, walking speed, double support time, nonparetic stance time, nonparetic step length, and paretic step length between the BBS high and BBS low groups (*p* < 0.05).

**Table 1:** Demographics of participants.

Variables	BBS high (n = 30)	BBS low (n = 14)	*p*
Age (years)	50.3 ± 13.2	56.4 ± 10.1	0.137
Sex (M:F)	23 : 7	12 : 2	0.500
Affected side (R:L)	13 : 17	8 : 6	0.405
Height (cm)	170.3 ± 7.1	170.1 ± 8.1	0.936
Weight (cm)	69.0 ± 8.4	72.3 ± 10.4	0.268
Onset (month)	9.2 ± 7.5	10.5 ± 11.2	0.660
Modified Ashworth scale (0:1:1 + :2)	2 : 8 : 19 : 1	0 : 3 : 11 : 0	0.373
Range of motion (degree)			
Dorsiflexion	15.9 ± 7.3	8.9 ± 9.3	0.009*
Plantarflexion	133.3 ± 9.8	131.4 ± 7.7	0.520
Inversion	24.0 ± 4.5	22.9 ± 4.2	0.456
Eversion	21.5 ± 3.5	19.2 ± 4.6	0.071
Isometric contraction force (N)			
Dorsiflexor	13.5 ± 4.8	9.7 ± 4.2	0.013*
Plantarflexor	15.3 ± 5.1	12.3 ± 4.9	0.072
Inverter	9.0 ± 2.3	6.8 ± 3.5	0.019*
Evertor	8.7 ± 4.6	6.2 ± 3.0	0.072
Fugl-Meyer (score)	18.4 ± 4.7	16.6 ± 2.2	0.180
Berg balance scale (score)	50.2 ± 2.5	39.3 ± 7.3	0.000*
Timed up and go (sec)	24.3 ± 12.5	40.8 ± 20.7	0.013*
Fall efficacy scale (score)	43.1 ± 29.2	62.3 ± 20.3	0.017*
Mini-mental state estimation (score)	28.3 ± 2.7	26.5 ± 3.0	0.047*
Spatiotemporal gait parameters			
Walking speed (cm/s)	50.7 ± 23.2	24.4 ± 13.5	< 0.001*
Cycle time (sec)	1.6 ± 0.3	1.8 ± 0.3	0.077
Double support time (s)	0.7 ± 0.3	1.1 ± 0.4	0.003*
Stride width (cm)	21.2 ± 3.9	22.9 ± 3.9	0.166
NP step length	34.8 ± 12.7	19.6 ± 12.8	0.001*
NP stance time (s)	1.2 ± 0.3	1.6 ± 0.5	0.002*
NP swing time (sec)	0.5 ± 0.3	0.4 ± 0.0	0.181
P Step Length (cm)	41.1 ± 14.0	22.4 ± 11.6	< 0.001*
P stance time (s)	1.0 ± 0.3	1.2 ± 0.2	0.074
P swing time (s)	0.5 ± 0.2	0.5 ± 0.1	0.389

Significant values are in bold.

The “*” symbol indicates significant differences between BBS high and BBS low groups (*p < 0.05).

*BBS* Berg balance scale, *NP* nonparetic, *P* paretic.

### Comparison PCs between two groups: main PCA results

Paretic PCA indicated that the initial 43 PCs accounted for over 99% of the joint movement pattern. Table 2 focused on the first 12 of these paretic PCs, each of which explained more than 1% of the total variance^19^. These PCs, together, explained 91.9% of the variance. The nonparetic PCA results indicated that 10 PCs explained 90% of the variance. Table 2 presents the explained variance, means, and standard deviations (SDs) of the PC scores by group. The univariate analysis (independent t-test) revealed significant differences between the BBS high and low groups on PC1, PC3, PC7, PC9, PC26, and PC38 in the paretic results (*p* < 0.15), explaining 56.7%, 5.8%, 2.8%, 1.8%, 0.2%, and 0.1% of the total variance, respectively. The nonparetic results revealed significant differences between the BBS high and low groups on PC1, PC2, PC3, PC5, PC6, PC7, and PC29 (*p* < 0.15), explaining 59.3%, 7.4%, 6.2%, 3.4%, 2.6%, 2.0%, and 0.2% of the total variance, respectively. Cohen’s *d* was used to compare the means and standard deviations between groups. There was a moderate to large effect for paretic PC1, PC3, PC6, PV9 and non-paretic PC1, PC2, PC3, PC4, PC5, PC6, PC7.

**Table 2:** Results of main principal component analysis.

Categories		Explained variance (%)	Cumulative (%)	BBS high (mean ± SD)	BBS low (mean ± SD)	*p*	Cohan's *d*
Paretic	PC1	56.7	56.7	− 33.5 ± 6.7	− 39.6 ± 12.6	0.107*	**0.605**
	PC2	7.1	63.8	0.1 ± 11.0	1.9 ± 17.2	0.728	0.125
	PC3	5.8	69.6	− 4.9 ± 8.5	5.6 ± 14.4	0.021*	**0.888**
	PC4	5.0	74.6	− 1.3 ± 10.6	3.7 ± 11.6	0.185	0.450
	PC5	3.9	78.4	− 0.3 ± 9.7	− 0.1 ± 10.0	0.935	0.020
	PC6	3.2	81.7	0.6 ± 9.3	− 0.7 ± 8.1	0.645	0.149
	PC7	2.8	84.5	− 2.4 ± 7.1	4.0 ± 9.2	0.032*	**0.779**
	PC8	2.2	86.7	− 0.8 ± 5.9	− 0.4 ± 9.9	0.887	0.049
	PC9	1.8	88.5	− 1.5 ± 6.0	2.1 ± 7.1	0.119*	**0.548**
	PC10	1.3	89.8	− 1.1 ± 5.3	1.7 ± 6.0	0.150	0.495
	PC11	1.1	90.9	0.4 ± 5.6	0.4 ± 4.5	0.996	0.001
	PC12	1	91.9	− 0.2 ± 5.6	− 0.3 ± 3.1	0.960	0.015
Non-paretic	PC1	59.3	59.3	− 35.1 ± 6.6	− 39.9 ± 8.8	0.087*	**0.612**
	PC2	7.4	66.7	− 2.8 ± 13.6	3.8 ± 12.3	0.121*	**0.510**
	PC3	6.2	72.9	− 2.9 ± 12.6	4.3 ± 10.5	0.057*	**0.623**
	PC4	5.5	78.4	2.9 ± 9.3	− 3.3 ± 14.5	0.161	**0.509**
	PC5	3.4	81.9	− 2.3 ± 8.9	2.8 ± 8.8	0.090*	**0.571**
	PC6	2.6	84.5	1.8 ± 7.4	− 2.6 ± 8.4	0.108*	**0.556**
	PC7	2.0	86.4	1.7 ± 7.2	− 2.5 ± 5.3	0.037*	**0.664**
	PC8	1.8	88.2	0.2 ± 6.2	− 1.3 ± 7.5	0.534	0.218
	PC9	1.3	89.6	0.6 ± 6.0	− 0.6 ± 5.2	0.514	0.214
	PC10	1.3	90.9	0.0 ± 5.9	0.2 ± 5.1	0.906	0.031

Significant values are in bold.

Principal component analysis was applied to the correlation matrix of 4848 variables (i.e., intra-participant mean, standard deviation, paretic side and nonparetic side for 101 time points, four angles in three axes) calculated from the 44 data sets (44 participants). The “*” symbol indicates significant differences between BBS high and BBS low groups (*p < 0.15). *PC* principal component, *BBS* Berg balance scale.

### Contributions of PC1 and PC2: the results of loading values of PCs

**Figure 1:**
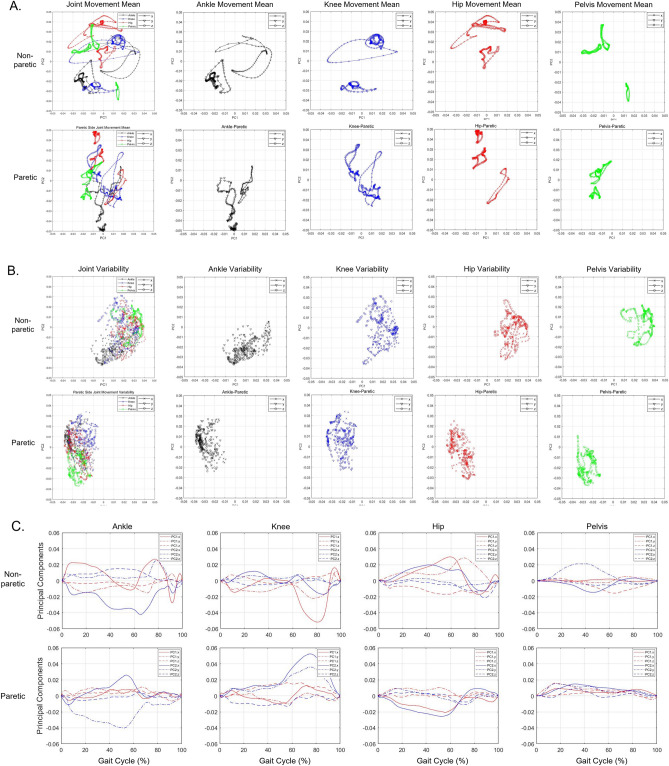
Loading values representing contributions to PC1 and PC2. Mean trajectory (**A**), distribution of variability (**B**), and time-varying trajectories of the loadings (**C**) of paretic and nonparetic PC1 and PC2 during a gait cycle, respectively.

Figure 1 presents the loading values, which describe the contribution of principal component (PC)1 and PC2 with 63.8% of the total variance. Figure 1A displays the mean loading values throughout the gait cycle. A higher absolute loading value indicates that the joint is the primary contributor during that particular gait cycle. The distribution of joint kinematics indicated that the PC1 loadings were greater in the nonparetic than in the paretic side, while the distribution of PC2 loadings was similar in both sides. The joint kinematics variability of PC1 and PC2 loadings on the nonparetic side was generally positively distributed, while the joint variability of loadings on the paretic side was generally negatively distributed (Fig. 1B). Principal joint movements were extracted from the gait cycle using absolute values of ± 0.2 of loading weights for each variable (Fig. 1C). The results for the nonparetic limb indicated that ankle dorsi-/plantarflexion (D/P) was significant throughout the entire gait cycle, particularly from mid-stance to mid-swing. Additionally, knee flexion/extension (F/E) was significant from initial swing to mid-swing, hip F/E was significant from pre-swing to initial swing, and hip abduction/adduction was significant from initial swing to mid-swing. Regarding the paretic limb, ankle D/P was significant from terminal stance to pre-swing, and ankle inversion/eversion was significant from mid-stance to pre-swing. In addition, knee F/E was significant from initial swing to mid-swing, knee varus/valgus was significant from initial swing to mid-swing, and hip F/E was significant from terminal stance to initial swing.

### Comparing gait kinematic characteristics: the results of reconstructed joint kinematic waveforms

**Figure 2:**
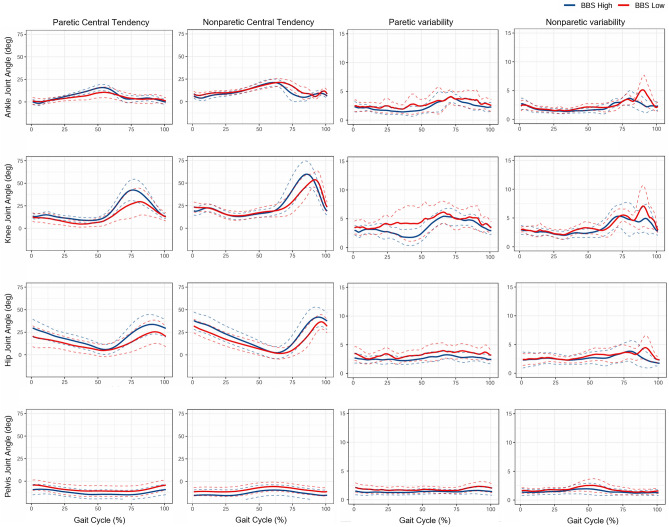
Waveforms of paretic central tendency (average), nonparetic central tendency, paretic variability (standard deviation) and nonparetic variability in sagittal planed reconstructed from the significant principal component score of principal component vectors between the two groups (Berg balance score high and low groups, *p* < 0.15).

Joint kinematic waveforms (mean and SD) of the pelvis, hip, knee, and ankle joint angles were reconstructed on the sagittal, frontal, and horizontal planes using the PCs that showed significant between-group differences (*p* < 0.15; Fig. 2). The reconstructed waveforms illustrated the differences between BBS high (blue line) and BBS low (red line) groups. The reconstructed waveforms for paretic central tendency revealed that the BBS low group exhibited smaller paretic ankle dorsiflexion during the stance phases and more ankle toe-in during all phases than the BBS high group. Additionally, the BBS low group displayed a smaller paretic knee flexion, particularly during the swing phase, and displayed a more flexed hip joint during the entire gait cycle. Moreover, their pelvic joints tended to posterior tilt and rotate more during the entire gait phase and upward rotate during the swing phase compared with the BBS high group. The reconstructed waveforms for nonparetic central tendency indicated that the BBS low group exhibited greater nonparetic ankle dorsiflexion during the swing phase and tended to exhibit more ankle eversion and toe-out during the entire gait phase than the BBS high group. Additionally, compared with the BBS high group, the BBS low group displayed smaller and delayed nonparetic knee flexion during the swing phase, more knee valgus during the mid-swing phase, more knee varus during the terminal swing and knee external rotations from terminal stance to mid-swing. Moreover, the nonparetic hip joints of the BBS low group showed smaller flexion and abduction during the entire gait phase compared with the BBS high group. Furthermore, the BBS low group exhibited more pelvic posterior tilt and downward and anterior rotation during the entire gait phase compared with the BBS high group. The study found significant differences in kinematic characteristics in both paretic and nonparetic limbs, particularly in the sagittal and horizontal planes, in relation to balance impairment. The waveforms reconstructed for paretic variability indicated that participants with a low BBS score exhibited greater paretic joint movement variability throughout the entire gait phase, particularly in the sagittal and horizontal planes, and greater and delayed nonparetic ankle, knee and hip variability in the sagittal plane during the terminal-swing phase.

### Principal component analysis accuracy: the predictive power of PCA

When the linear support vector machine (SVM) model was used to predict the BBS high group, the f1-score, representing the harmonic mean of precision and recall, was the highest for both paretic and nonparetic at 0.880 and 0.857, respectively. The area under the curve for paretic and nonparetic was 0.889 and 0.861, respectively (Supplementary Information Table S3).

### Key functional characteristics: the results of factor analysis

The exploratory factor analysis suggested that three factors could be extracted (Supplementary Information Table S1). The first factor predominantly explained patients’ balance and gait functions, explaining 86.3% of the variance. The second factor was significantly related to temporal gait variables, explaining 82.2% of the variance. The third factor was the range of motion of dorsiflexion during gait, which explained 94.4% of the variance in the data. The primary determinants of physical functioning in patients with stroke were balance ability and gait function, which encompass gait speed and spatiotemporal gait parameters. Both the paretic and nonparetic sides are required to contribute to these factors.

### Principal components and clinical variables: relationship between PCs and clinical variables

Pearson’s linear correlation coefficients between the clinical and spatiotemporal gait parameters and PCs are presented in Supplementary Information Table S2. Paretic PC1 and nonparetic PC1 similarly correlated with paretic sensation, fall efficacy, balance ability, bilateral gait parameters (cycle time, double support time, walking speed, and step length), and nonparetic swing time (*p* < 0.05). Conversely, paretic PC2 was associated with paretic sensorimotor function, including the range of motion of paretic dorsiflexion and paretic sensation while nonparetic PC2 was significantly associated with paretic motor function, fall efficacy, balance, and several spatiotemporal gait parameters (*p* < 0.05).

## Discussion

This study utilised PCA to create a streamlined stroke-specific gait model and differentiated it according to balance impairment. In particular, patients with strokes, both paretic and nonparetic, exhibited differences in kinematic characteristics during gait in response to balance impairment. Furthermore, the group with balance impairment exhibited an increase in joint variability on the paretic side, particularly in the sagittal and horizontal planes, as well as an increase in ankle and knee variability on the nonparetic side in the sagittal plane.

**Figure 3:**
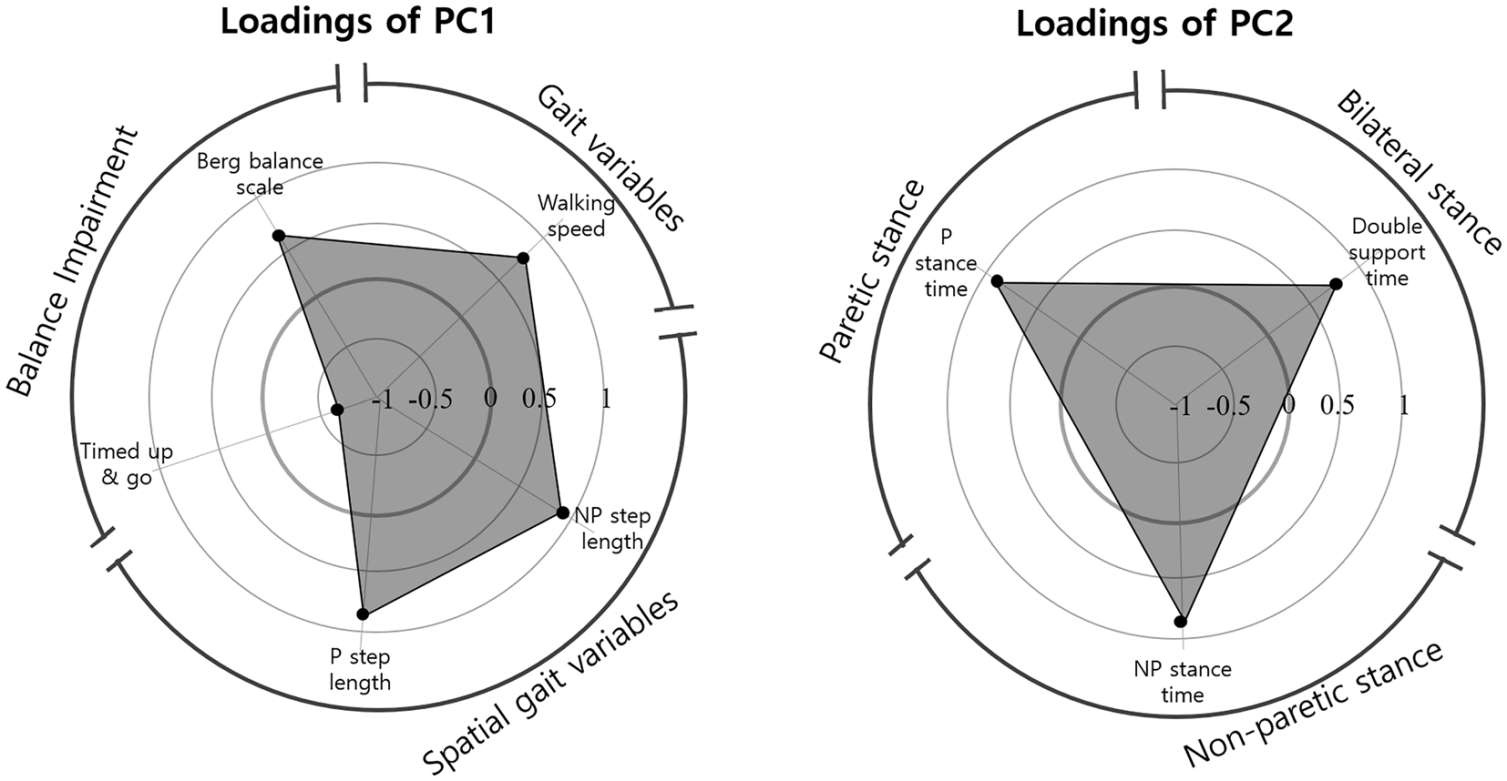
Loadings of PC1 (left) and PC2 (right) for walking and balance characteristics of patients with stroke.

Asymmetrical balance control and gait deviations are frequently observed after a stroke. This study’s factor analysis results indicated that balance ability, walking speed, and bilateral step length were the primary factors affecting the physical function of patients who had experienced a stroke (Fig. 3). Impaired balance can limit an individual’s ability to perform activities that involve their feet, such as standing, walking, carrying objects, grocery shopping, toileting, and travelling to socialize with their friends, and represents one of the largest contributing factors to falls in individuals who have experienced a stroke^6,7^. Previous studies have demonstrated that the BBS associated with walking speed, paretic step length, and swing time asymmetry in patients with strokes, suggesting spatiotemporal gait asymmetry, was more closely related to balance measures involving dynamic tasks than static tasks^2,20^. Moreover, the cut-off BBS score of 29 on admission predicted that an individual would go on to achieve community walking speed (n = 123, area under the curve = 0.88, 95% confidence interval [CI] 0.81–0.95), and the cut-off score of 12 predicted a non-ambulator to regain unassisted ambulation (n = 84, AUC 0.73, 95% CI 0.62–0.84)^21^. A recent study found that balance ability, as measured by the BBS, was a significant predictor of affected leg extension angle during gait in stroke patients (p < 0.001)^22^. They also suggested that increasing balance ability is useful in increasing leg extension angle during walking and may be effective in improving walking speed^22^. Therefore, gait performance with compensatory adaptations of patients with strokes was associated with balance deficit and the relationship of these factors in the physical functioning of these patients requires consideration. Gait performance and balance impairments may be associated with similar impairments, such as hemiparesis, altered sensory function, or lack of confidence in the paretic limb^23^.

This study’s results allow for the comparison of paretic and nonparetic differences according to balance impairment. Two inferences can be drawn from the PCA results. First, the kinematic difference of both paretic and nonparetic limbs in relation to balance impairment. The BBS low group with balance impairment was accompanied by not only paretic (PC1, PC3, PC7, PC9, PC26, and PC38) but also nonparetic (PC1, PC2, PC3, PC5, PC6, PC7, PC12, and PC29) kinematic differences compared with the BBS high group (*p* < 0.15). This study found significant differences in kinematic characteristics in both paretic and nonparetic limbs, particularly in the sagittal and horizontal planes, in relation to balance impairment. Do stroke patients experience critical changes in their nonparetic side as well as their paretic side? Balancing and walking often require simultaneous use of both lower extremities. Although stroke patients commonly experience difficulties in all planes, particularly in the direction of their paretic limb, loading the nonparetic limb may also pose a challenge^24,25^. Generally, asymmetrical postural control to maintain the centre of mass toward the nonparetic limb is observed during gait in individuals who have experienced a stroke^26,27^. Spatiotemporal asymmetry is a common characteristic of patients with strokes in gait kinematic analyses such as asymmetry in stance time, single stance time, double support time, swing time, and step length^1,9,12,28^. The PC1 and PC2 loading results in this study may have been more heavily weighted towards the nonparetic limb because of this biased gait. These associations between uneven weight distribution during gait and various spatiotemporal gait asymmetries in patients with strokes have frequently been reported^27,29^. However, the function of the nonparetic limb is influenced by paretic kinematics and sometimes plays a significant role in stroke gait. The gait kinematics of both lower limbs are affected by slightly different causes. In this study, both paretic PC1 and nonparetic PC1, which accounted for 56.7% and 59.3% of the total variance, respectively, were associated with balance measures (BBS and TUG), spatiotemporal gait parameters, paretic sensation, and fall efficacy (*p* < 0.05). However, paretic PC2 (7.1% of total variance) was associated with the paretic range of motion of dorsiflexion, sensation, and balance measures, while nonparetic PC2 (7.4% of total variance) was associated with the paretic strength of the plantarflexor, fall efficacy, and gait and balance variables (*p* < 0.05). These results suggest that the distinct kinematics of the paretic and nonparetic limbs may be attributed to slightly different causes.

Second, the kinematic characteristics of both lower limbs utilise significant PCs during a gait cycle in relation to balance impairment. The ability to balance may exacerbate the imbalance of the kinematic control of both limbs. A higher degree of spatiotemporal asymmetry is associated with greater balance impairments, such as a lower BBS score or a larger step width^2^. Several significant relationships were identified between BBS scores, step length, and swing time asymmetries (*r* = − 0.36 to − 0.63)^2^. This is related to the decreased contribution of anterior/posterior balance control of the paretic limb^8^. Common kinematic deviations in the paretic limb involve sagittal plane disturbances of hip, knee, and ankle motions. These include limited or reduced hip and knee flexion, as well as reduced ankle dorsiflexion or continuous plantar flexion^30^. In this study, participants with balance impairment exhibited distinct kinematic characteristics during swing phase in the sagittal plane compared with those without balance impairment. These differences were identified through the level of loadings with significant PC contributions and gait cycle-specific joint kinematics retransformed into significant PCs compared between the two groups. Specifically, they displayed smaller paretic ankle dorsiflexion from mid-stance to initial-swing, smaller knee flexion from initial swing to mid-swing, and smaller hip flexion from pre-swing to initial swing. Additionally, nonparetic joint kinematics showed greater ankle dorsiflexion, smaller knee flexion, and smaller hip flexion from initial swing to mid-swing. Previous studies indicated that reduced paretic contribution, specifically decreased kinematic movement in the sagittal plane, was associated with spatiotemporal gait symmetry, including step length, stance time, and swing time^8,31^. During gait, the duration of the paretic stance is decreased owing to impaired control of forward progression during single support on the paretic limb compared with the nonparetic limb, compensating for the decreased paretic stance time^32^. These compensatory adaptations typically affected the low maximum excursion of hip extension, ankle plantarflexion during the stance phase, low maximum excursion of hip and knee flexion and ankle dorsiflexion during the swing phase, and excessive pelvic tilts during the stance and swing phases^33^. According to a recent study, reduced knee flexion during the pre-swing and swing phase is associated with paretic ground reaction force in the late stance phase^34^. This relationship is related to the cooperation between the paretic and non-paretic thighs^34^. In this study, the BBS low group exhibited an increase in joint variability on the paretic side, particularly in the sagittal and horizontal planes, as well as an increase in ankle and knee variability on the nonparetic side in the sagittal plane. Variability changes were quantifiable markers of post-stroke gait^35^. Patients with strokes with severe gait impairments and slow walking speed exhibited significant temporal variabilities and asymmetry^35,36^. Therefore, the increase in kinematic variability in the BBS low group, both paretic and non-paretic, particularly in the sagittal plane, may have contributed to gait deficits, such as a significant decrease in walking speed. However, a recent study found that stroke severity did not always correlate with slower gait speed^37^. The study also indicated individuals with mild motor paralysis and slow walking speed demonstrated decreased trunk stability and increased paretic co-contraction of the shank muscles^37^. These results indicate that stroke severity, which is related to balance impairment, affects gait strategy and should be considered in conjunction with physical function assessments and specific gait analyses, such as PCA.

The novelty of the present study lies in its utilisation of PCA to reveal the kinematic characteristics of the paretic and nonparetic sides in patients who had experienced a stroke according to balance impairment. In conclusion, the present study showed that the differences in joint kinematics that can be distinguished by balance impairment are both paretic and nonparetic changes in joint kinematics of ankle, knee, and hip and increased joint variability in the sagittal plane during the swing phase. The predictive power of PCA with the linear SVM model was significantly higher than 88% for paretic limbs and 85.7% for nonparetic limbs. Therefore, this study is expected to provide a more comprehensive understanding of stroke hemiparesis gait patterns and suggest more effective gait rehabilitation interventions according to balance impairment as a validated result of the proposed novel PCA method. In clinical practice, which typically focuses on problems in the paretic lower limb, this study suggest that it is important to consider nonparetic limb function and bilateral coordination. Furthermore, PCA may be a useful assessment tool to distinguish differences in balance impairment and to diagnose, assess and monitor dynamic symmetry during gait in patients with stroke.

However, this study had several limitations. First, the distribution of the two groups, divided by balance impairment, was unbalanced. Second, this study only included participants who had sufficient walking ability to not require an assistive device. This may impact the interpretation of the study's findings as it could affect gait symmetry, among other factors. Third, other clinical factors that may affect gait function, such as metabolic cost, stroke severity, endurance, activity level, and social participation, were not measured. Fourth, the PCA did not include muscle activation and kinetics, which are significant explanations for gait. Fifth, gait was only measured at a comfortable walking speed. Future studies should consider analysing the gait characteristics of patients with strokes via PCA in different walking conditions such as various walking speeds, walking over obstacles, walking on uneven/slippery surfaces, and external perturbations in relation to falls.

## Methods

### Participants

A total of 45 individuals with chronic strokes were recruited from in-patient clinics at National Rehabilitation Centre, Seoul, South Korea to voluntarily participate in the study. All participants were right-side dominant and had hemiplegic gait patterns that affected their postural balance and ambulation. Inclusion criteria included individuals who had (1) experienced a stroke more than six months prior, (2) the ability to walk (with or without assistive devices) independently for at least 10 m in a straight line, (3) the absence of orthopaedic problems or significant pains in the lower extremities, and (4) hemiplegia in the right or left sides of the body. Patients with poor visual depth perception, inability to control their posture or limbs (Modified Ashworth Scale score > 3), and cognitive deficits that influenced their ability to understand the study and follow instructions (Mini-mental state examination < 20) were excluded. The experimental protocol was approved by the ethical review board at the National Rehabilitation Centre and all participants provided written informed consent before participating (IRB number: NRC 2022-02-014, 29/03/2022). The research complied with the principles of the Declaration of Helsinki.

### Definition of balance impairment

Balance is one of the parameters that predict performance in activities of daily living. The BBS is one of the most widely used assessment tools for balance and includes multiple items examining different aspects of balance performance^38^. A cutoff score of < 45 in the BBS can be successfully used to identify risk of falling among community-dwelling older adults^39^. In the stroke population, BBS cutoff scores have been determined to predict the risk of falls, length of stay and discharge destination in inpatient rehabilitation, and degree of improvement to achieve community walking speed^21,38^. However, other studies have reported that BBS alone cannot assess mobility and falls, and demonstrates poor prediction of falls after a stroke^40^; therefore, using a combination of the BBS and the TUG test, which has been shown to predict balance impairment after a stroke, is recommended^41^. The TUG test was used to assess mobility, as well as both static and dynamic balance, and suggested that a TUG time of ≥ 13.5 s can be classified as indicative of balance impairment^42^. Owing to the acceptable sensitivity (91% and 80%) and specificity (82% and 100%) for the BBS and the TUG test, respectively, to predict the risk of falling^39,42^, a combined cutoff score of BBS < 45 and TUG ≥ 13.5 s was defined as indicating balance impairment in this study. When participants were divided into two groups based on balance impairment criteria, those meeting the BBS score criteria also met the TUG criteria. Therefore, the groups in this study were named the BBS high group and the BBS low group.

### Clinical assessments

All measurements were performed by skilled physiotherapists. As an assessment of ankle function, the passive range of motion (ROM), of the paretic ankle was measured using a portable goniometer. The average values of three measurements were recorded for the maximum passive ROM of dorsiflexion, plantar flexion, eversion, and inversion. To measure ankle muscle strength, the isometric contraction force of the paretic ankle muscle was measured using a portable manual muscle strength tester. The isometric strength of the ankle dorsiflexor, plantar flexor, invertor, and evertor was measured for 5 s and the maximum value was recorded. The motor domain of the Fugl-Meyer lower extremity (FM-L) assessment was used to measure motor impairment^43^. This domain includes measurements of movement, coordination, and reflex action for the hip, knee, and ankle. The FM-L is rated on a three-point ordinal scale (0 = cannot be performed, 1 = partially performed, and 2 = fully performed). The maximum possible score for the motor domain of the FM-L assessment is 34, corresponding to full sensorimotor recovery. The Korean version of the fall efficacy scale was utilised to ascertain the patient’s level of confidence in performing daily living activities^44^. This self-report questionnaire contains 10 items, each scored on a scale of 0–10. The total summed score ranges from 0 to 100, with a higher score indicating increased confidence in performing daily living activities without falling.

### Gait measurements

Gait measurement was performed in a room with a straight 10-m. During gait measurement, participants were asked to walk at a comfortable, self-selected speed. Three-dimensional (3D) positional data were measured during walking, using reflective markers and a 12-camera 3D motion capture system (VICON, Saint Helens, UK) with a 100 Hz sampling frequency. A total of 23 reflective markers were attached following the guidelines of the Visual 3D software (C-Motion Ubc., Rockville, MD, US). Before the walking trials, the positions of the markers were recorded while the participants were stationary. Subsequently, the participants were supplied with sufficient walking practice to ensure a natural gait. After the practice, three successful standing and gait trials were recorded per leg.

### Sample size analysis

The sample size was calculated as described in a previous study that identified biomechanical features of gait waveform data associated with knee osteoarthritis^45^. The calculation was based on the independent t-test value for between-group comparison of principal components resulting from principal component analysis performed using an acceptable level of significance of 0.05 at 95% power. The total sample size was determined to be fourteen for each group (expected effect size: 1.321; actual power: 0.951). Therefore, a total of 45 participants were recruited for the study, taking into account the power of analysis, variables, and a 30% adjustment for dropout rates during study conduct and missing values during analysis. The G ∗ Power 3.1.9.2 program was used.

### Data analysis

The raw motion data were digitally filtered using a zero-lag, fourth-order, low-pass Butterworth filter; the filter cut-off frequency was 6 Hz. The hip, knee, and ankle joint angles, and the pelvis-link angle during one gait cycle were calculated for the x-axis (i.e., flexion–extension), y-axis (i.e., abduction–adduction), and z-axis (i.e., internal–external rotation) using a Cardan sequence of rotations (X–Y–Z) from the trajectories measured in each trial (joint-specific movements for each axis are presented in Supplementary Information Table S4). Each angle was time-normalized using the gait cycle duration and divided into 101 variables ranging from 0 to 100%^46^. Therefore, each trial corresponded to a dataset of 4848 variables (101 time points, four angles in three axes, means, SDs, and two types of variables: paretic and nonparetic side). Average and within-participant coefficients of variation of walking speed, cycle time, double support time, stride width, step length, stance time, and swing time were determined to help understand the gait characteristics. Low-pass filtering, variable calculation (i.e., joint and link angles and spatiotemporal parameters), and time normalization processes were performed using Visual 3D software.

### Principal component analysis

PCA was applied to the correlation matrix of the 4848 variables calculated from the 132 data points (44 participants in three trials). The specific PCA procedure was as follows. First, intra-participant average and SDs were calculated for each time point within the three trials of data obtained from each participant. Second, mean centring was conducted on each of the 2424 variables (i.e., averages and SDs for 101-time points, four angles in three axes) for each paretic side and nonparetic side using the z-score:

$${Z}_{t}=({X}_{t}-{\mu }_{t})/{\sigma }_{t}$$

where ${Z}_{t}$ refers to the z-score for the parameter t, ${X}_{t}$ refers to the raw data of the parameter t, ${\mu }_{t}$ refers to the mean of the parameter t for the participant, and ${\sigma }_{t}$ refers to the SD of the parameter t. The value of the parameter t ranged between 1 and 2424. Third, input matrices of 44 data points (44 participants) by 2424 variables were constructed for both paretic and nonparetic sides. Fourth, PC vectors (PCVs) were extracted until their cumulative ratio attained 91.9% for the paretic side and 90.0% for the nonparetic side of the total variance and statistical analyses were conducted to identify the main effects of balance impairment on the joint kinematic characteristics during gait represented by the PCVs. Fifth, joint kinematic waveforms were reconstructed from the significant PC scores with significant differences between the two groups according to balance impairment (*p* < 0.15), to interpret data on the average joint angle and joint angle variability corresponding to the PCVs. Sixth, the PC1 and PC2 loading values, which had the largest variation, were plotted to show their variation during the gait phase. The magnitude of these loading values determined their contribution to the PC. Finally, to investigate the predictive power of the PCA results, a forecast model was created for the BBS high group, using only PCs with a *p*-value lower than 0.15. The analysis involved implementing various models, including RandomForest, linear SVM, Polynomial SVM, XGBoost, and logistic models. The model was developed using Python 3.6.3.

### Statistical analysis

All statistical analyses were performed using SPSS version 22.0 (IBM, Armonk, NY, USA). The baseline data’s normal distribution was assessed using the Shapiro–Wilk test. The independent t-test or chi-square test was conducted to compare the BBS high and low groups at baseline. Factor analysis was conducted to explore the underlying structure of the relationships with clinical assessments and spatiotemporal gait parameters. Bartlett’s test for dimensionality indicated significance (*p* < 0.001) and the communality indicated that all parameters exceeded 0.5; therefore, all components could be used for factor analysis. Based on the Scree plot and the result of the rotated component material, the three-factor solution was considered further. Univariate analysis was conducted on the PCs between the two groups (BBS high and BBS low) using independent t-tests (*p* < 0.15), in the same way that the differences between the PCVs of different groups were analysed in previous studies^14,15^. Furthermore, Cohen’s *d* effect size was calculated to validate the results of the t-test and the results were interpreted using 0.2 for a small effect, 0.5 for a moderate effect, and 0.8 for a large effect. Additionally, Pearson’s linear correlation coefficients were determined between the PCs and clinical assessments (*p* < 0.05).

### Supplementary Information

Supplementary Information.

## Data Availability

Supplementary information is available for this study. The datasets generated and/or analysed during this study are available from the corresponding author upon reasonable request.
