# Supplementary material for: Stroke walking and balance characteristics via principal component analysis
Source: Sci Rep. 2024 May 7;14:10465. doi: 10.1038/s41598-024-60943-5 (PMC11076567; doi:10.1038/s41598-024-60943-5)
Supplement: Supplementary file 1 — Supplementary Information. [file 41598_2024_60943_MOESM1_ESM.docx]

**Supplementary Information**

**Table 1.** Exploratory factor analysis to explore the underlying structure of the relationship with clinical assessments and spatiotemporal gait parameters: three-factor rotated solution (varimax rotation).

| **Categories** | **Item** | **Factor 1** | **Factor 2** | **Factor 3** | **Cronbach Alpha** |
| --- | --- | --- | --- | --- | --- |
| Balance & Gait  (Spatial variables) | Berg balance scale | 0.629 |  |  | -1.063 |
|  | Timed up and go | -0.699 |  |  |  |
|  | Walking speed | 0.850 |  |  |  |
|  | Nonparetic step length | 0.915 |  |  |  |
|  | Paretic step length | 0.863 |  |  |  |
| Gait (Temporal variables) | Paretic stance time |  | 0.934 |  | 0.933 |
|  | Nonparetic stance time |  | 0.922 |  |  |
|  | Double support time |  | 0.822 |  |  |
| Joint range of motion | Dorsiflexion during gait |  |  | 0.944 | - |

KMO=0.655, Chi-square=432.612, df(p)=36(0.000)

**Table 2.** Correlation coefficients between the clinical variables and principal components (PCs). We analyzed Pearson's linear correlation coefficients between PCs and other clinical assessments and spatiotemporal gait parameters. The “*” symbol indicates significant differences between BBS high and BBS low groups (**p* < 0.05). *DF* dorsiflexion, *PF* plantarflexion, *PC* principal component.

| **Categories** | | **Items** | **Paretic** | | **Nonparetic** | |
| --- | --- | --- | --- | --- | --- | --- |
|  |  |  | **PC1** | **PC2** | **PC1** | **PC2** |
| Sensorimotor | Paretic | Range of motion of DF | 0.184 | **-0.424*** | 0.210 | -0.035 |
|  |  | Strength of PF | 0.220 | 0.192 | -0.091 | **-0.343*** |
|  |  | Sensation | **-0.389*** | **0.352*** | **-0.379*** | 0.187 |
| Balance | Bilateral | Berg balance score | **0.459*** | -0.234 | **0.431*** | -0.251 |
|  |  | Timed up and go | **-0.315*** | **0.330*** | **-0.512*** | **0.319*** |
| Confidence | - | Fall efficacy scale | **-0.447*** | -0.263 | **-0.424*** | **0.371*** |
| Gait | Bilateral | Walking Speed | **0.538*** | -0.021 | **0.603*** | **-0.418*** |
|  |  | Double Support Time | **-0.598*** | -0.035 | **-0.616*** | **0.500*** |
|  |  | Cycle Time | **-0.368*** | -0.131 | **-0.332*** | **0.391*** |
|  | Paretic | Step Length | **0.529*** | -0.158 | **0.619*** | **-0.373*** |
|  |  | Stance Time | -0.280 | -0.060 | -0.227 | **0.326*** |
|  | Nonparetic | Step Length | **0.530*** | -0.033 | **0.571*** | **-0.370*** |
|  |  | Stance Time | **-0.489*** | -0.111 | **-0.500*** | **0.484*** |
|  |  | Swing Time | 0.248 | -0.186 | **0.342*** | -0.157 |

**Table 3.** Results of the prediction model indicate significant differences (p < 0.15) in group comparison for PCs. The linear Support Vector Machine model had the highest f1-score of 0.880 and 0.857 for paretic and nonparetic, respectively. Additionally, it showed the highest AUC of 0.889 and 0.861 for paretic and nonparetic, correspondingly. *SVM* Support Vector Machine, *AUC* Area under the Curve.

| **Model** | **Side** | **f1-score** | **AUC** | **Accuracy** | **Precision** |
| --- | --- | --- | --- | --- | --- |
| RandomForest | Paretic | 0.833 | 0.750 | 0.778 | 0.830 |
|  | Nonparetic | 0.828 | 0.583 | 0.722 | 0.710 |
| **SVM(linear)** | **Paretic** | **0.880** | **0.889** | **0.833** | **0.850** |
|  | **Nonparetic** | **0.857** | **0.861** | **0.778** | **0.750** |
| SVM(Poly) | Paretic | 0.800 | 0.847 | 0.666 | 0.670 |
|  | Nonparetic | 0.857 | 0.791 | 0.778 | 0.750 |
| XGBoost | Paretic | 0.833 | 0.750 | 0.778 | 0.830 |
|  | Nonparetic | 0.692 | 0.458 | 0.556 | 0.640 |
| logistic | Paretic | 0.880 | 0.792 | 0.833 | 0.850 |
|  | Nonparetic | 0.857 | 0.667 | 0.778 | 0.750 |

**Table 4.** Definition of joint-specific motion for each axis of the kinematic data used in principal component analysis.

| **Categories** | | | **Joint movements** | | | |
| --- | --- | --- | --- | --- | --- | --- |
| Plane | Axis | Direction | Pelvis | Hip | Knee | Ankle |
| Sagittal plane | X | + | Posterior tilt | Flexion | Flexion | Dorsiflexion |
|  |  | - | Anterior tilt | Extension | Extension | Plantarflexion |
| Frontal plane | Y | + | Upward rotation | Adduction | Varus | Inversion |
|  |  | - | Downward rotation | Abduction | Valgus | Eversion |
| Horizontal plane | Z | + | Posterior rotation | Internal rotation | Internal rotation | Toe in |
|  |  | - | Anterior rotation | External rotation | External rotation | Toe out |
